# Dietary resveratrol improves meat quality and antioxidant capacity of Tibetan sheep by regulating muscle fiber characteristics and mitochondrial function

**DOI:** 10.3389/fnut.2026.1905065

**Published:** 2026-08-14

**Authors:** Xiongxiong Li, Zengkui Lu, Chao Yuan, Chen Zheng, Ting Liu, Bowen Chen, Jing Wang, Jianbin Liu

**Affiliations:** 1 Key Laboratory of Animal Genetics and Breeding on Tibetan Plateau, Ministry of Agriculture and Rural Affairs, Lanzhou Institute of Husbandry and Pharmaceutical Sciences of Chinese Academy of Agricultural Sciences, Lanzhou, China; 2 Sheep Breeding Engineering Technology Research Center of Chinese Academy of Agricultural Sciences, Lanzhou, China; 3 College of Animal Science and Technology, Gansu Agricultural University, Lanzhou, China

**Keywords:** mitochondrial function, muscle antioxidant capacity, muscle fiber type transformation, resveratrol, Tibetan sheep

## Abstract

**Objective:**

Combining metabolomics and proteomics, this study aimed to investigate the effects of resveratrol (RES) on meat quality, antioxidant capacity, muscle fiber characteristics and mitochondrial function in Tibetan sheep.

**Methods:**

Thirty healthy, 10-month-old male Tibetan sheep (33.87 ± 1.65 kg) were divided into three treatments: CON (basal diet), ResL (basal diet + 80 mg/kg RES), and ResH (basal diet + 360 mg/kg RES; *n* = 10). The experiment had a prefeeding period of 10 d and a treatment period of 84 d.

**Results:**

Dietary RES supplementation significantly improved meat quality by decreasing shear force and drip loss, and increasing crude protein content (*P* < 0.05). For antioxidant capacity, RES enhanced SOD and GSH-Px activities (*P* < 0.05), upregulated *SOD1, SOD2*, and *Nrf2* (*P* < 0.05), and downregulated *Keap1* expression (*P* < 0.01). Regarding muscle fiber traits, RES promoted slow-twitch fiber proportion, upregulated *MyHC IIa, IGF-1* and *mTOR*, while downregulating *MyHC IIx* and *FoxO3* expression (*P* < 0.05). For metabolic regulation, RES did not alter lactate or glycogen levels (*P* > 0.05), but increased glucose content, reduced pyruvate kinase (PK) activity, and enhanced hexokinase (HK), succinic dehydrogenase (SDH), NAD-dependent malate dehydrogenase (NAD-MDH), and mitochondrial respiratory chain complex (MRCC) II/III activities, along with upregulating *NRF1* and *TFAM* expression (*P* < 0.05). Metabolomic and proteomic analyses revealed that key proteins (PKM, PFKM, PGM1, PGAM2, HADHB, NDUFA8, NDUFS4, and GOT2) and differential metabolites (4-methylene-L-glutamine, anserine, betaine aldehyde, and N1-Methyl-2-pyridone-5-carboxamide) were closely correlated with muscle antioxidant capacity, mitochondrial function and meat quality (*P* < 0.05).

**Conclusion:**

In summary, RES collaboratively regulates muscle energy metabolism, muscle fiber type transformation and antioxidant capacity by modulating key glycolytic and mitochondrial respiratory chain proteins, as well as critical metabolites, ultimately enhancing the meat quality of Tibetan sheep.

## 1 Introduction

Tibetan sheep are a dominant livestock breed on the Tibetan Plateau and provide a crucial source of meat and livelihood for local herders (1). Their meat is highly favored by consumers for its tender texture, high protein, and low fat characteristics (2). Nevertheless, due to traditional grazing patterns (3), seasonal nutritional variation in pastures (4), and plateau oxidative stress (5), the meat quality of Tibetan sheep is impaired by nutritional imbalance, oxidative muscle damage, and altered fiber composition. Previous studies have found that natural plant extracts can effectively enhance muscle antioxidant capacity and improve meat quality (6, 7).

Resveratrol (RES) is a natural polyphenolic compound widely found in plants such as grapes, pomegranates, and peanuts, and it exhibits multiple biological functions, including antioxidant, anti-inflammatory, and metabolic regulatory effects (8). Previous studies have confirmed that dietary RES supplementation can enhance muscle antioxidant capacity and meat quality in pigs (9), goats (10), and broilers (11), while also increasing myoglobin content and maintaining muscle color stability in beef (12). RES enhances muscle antioxidant capacity by activating the Nrf2/Keap1 pathway, which initiates the transcription of downstream antioxidant genes such as superoxide dismutase (*SOD*) and glutathione peroxidase (*GSH-Px*), thereby further enhancing free radical scavenging capacity and oxidative stress tolerance (13). In addition, RES also modulates muscle fiber type transformation by increasing the proportion of slow-twitch oxidative fibers, thereby enhancing the muscle's oxidative metabolic potential (14).

Muscle fiber type is a key intrinsic factor determining meat quality. Based on myosin heavy chain (MyHC) isoforms, muscle fibers are primarily classified into slow-twitch oxidative (Type I), fast-twitch oxidative (Type IIa), and fast-twitch glycolytic (Type IIb/IIx) types (15). Muscles with a high proportion of oxidative muscle fibers typically exhibit better tenderness, meat color stability, and water-holding capacity (16). Research indicates that metabolic enzyme activities are closely related to muscle fiber type; the activities of succinate dehydrogenase (SDH) and malate dehydrogenase (MDH) are higher in oxidative muscle fibers, whereas lactate dehydrogenase (LDH) activity is higher in glycolytic muscle fibers (17). Mitochondrial biosynthesis and function are pivotal for governing the oxidative metabolic capacity of muscle fibers, with nuclear respiratory factor 1 (*NRF1*) and mitochondrial transcription factor A *(TFAM*) serving as core regulators of mitochondrial DNA replication and transcription (18).

Despite the well-documented benefits of RES for meat quality and antioxidant capacity in various species, its effects in Tibetan sheep remain largely unexplored. In particular, no studies have investigated how RES modulates meat quality via regulating metabolic enzyme activity, mitochondrial function, and muscle fiber type transformation in this breed. Therefore, the present study systematically evaluated the effects of dietary RES supplementation on meat quality, antioxidant capacity, muscle fiber characteristics, skeletal muscle development, energy metabolism, and mitochondrial function in Tibetan sheep. Integrated metabolomic and proteomic analyses were further employed to elucidate the underlying molecular mechanisms. Our findings are expected to provide a theoretical foundation for the application of RES as a feed additive in plateau sheep production and to offer a novel strategy for meat quality improvement.

## 2 Materials and methods

### 2.1 Resveratrol

RES was purchased from Xi'an Fenghe Biotechnology Co., Ltd., China, with a purity of 98%. It is a white to pale yellow powder, insoluble in water, easily soluble in organic solvents such as ethanol, with no special odor.

### 2.2 Animals, diets, and experimental design

A total of 30 healthy 10-month-old male Tibetan sheep of similar weight (33.87 ± 1.65 kg) were divided into three dietary treatments: CON (basal diet), ResL (basal diet + 80 mg/kg RES), and ResH (basal diet + 360 mg/kg RES). The experimental sheep in each group were housed in individual pens, with 10 sheep per pen; each sheep serving as one biological replicate (*n* = 10 per group). The experiment had a prefeeding period of 10 d and a treatment period of 84 d. Tibetan sheep were housed on a large-scale, well-managed ranch in Xiahe County, Gansu Province, China, in a temperature-controlled facility with adequate lighting, ventilation, and access to an exercise yard. The experimental diets were a total mixed pellet diet formulated by the Agricultural Industry Standard Mutton Sheep Feeding Standard of the People's Republic of China (Table 1), and Tibetan sheep were fed full mixed pellet feed twice daily (08:00 and 17:00), with *ad libitum* intake and free access to water through fresh tap water. For feed formulation, RES was uniformly incorporated into the basal diet at concentrations of 80 and 360 mg/kg to formulate total mixed pellet diets. After the experiment, six lambs per group were randomly selected for slaughter in accordance with GB 12694-2016 after 12 h without feeding. Selection adhered to random principles to ensure the chosen individuals were representative of the overall population level.

**Table 1:** Basic diet formulation.

Feed type	Items	Diets/%	Nutrient levels	
Roughage	Alfalfa hay	8.00	Metabolizable energy/(MJ/kg)	12.01
	Corn stover	5.00	H_2_O/%	10.65
	Sunflower husk	5.00	Crude protein/%	15.23
	Soybean hulls	3.00	Crude Ash/%	8.91
	Corn hulls	10.00	Crude fat/%	5.74
Concentrate	Corn	46.21	Crude fiber/%	3.26
	Soybean meal	7.65	Calcium/%	0.56
	Safflower seed meal	3.50	Digestible P/%	0.25
	Corn germ meal	3.00		
	Molasses	3.00		
	Cottonseed meal	2.50		
	Rapeseed meal	0.82		
	CaHPO4	1.14		
	NaHCO3	1.00		
	Salt	0.68		
	Premix^a^	0.50		

^a^Each kilogram of the premix provided the following: VA, 10000 IU; VD3, 5000 IU; VE, 50 IU; Cu, 30 mg; Fe 100 mg; Mn, 30 mg; Zn, 75 mg; I, 0.5 mg; and Se, 0.4 mg.

### 2.3 Meat quality analysis

After the Tibetan sheep had been slaughtered, the muscle samples of the left longissimus dorsi (LD) muscle (12th and 13th ribs) were used to determine shear force and drip loss. One part (approx. 5 g) was collected quickly and placed in liquid nitrogen for further analysis. After that, the muscles were placed in a 4°C cooler for 24 h for meat quality analysis.

Association of Official Analytical Chemists methods (AOAC, 2005) were used to assess intramuscular fat and crude protein content. The shear force was determined using a meat tenderness tester. A thermometer was inserted into the muscle wrapped in a polyethene bag. After the water bath temperature reached 70°C, the muscle sample was taken out and cooled to 35°C, and the shear force value was measured perpendicular to the muscle fiber direction. Mutton drip loss was sampled along the muscle fiber direction, weighed (W1), and then the meat strips were suspended in plastic bottles and placed at 4°C for 24 h. The meat strip weight (W2) was measured and calculated according to the following formula.


$$\begin{array}{l} Driploss\left(\%\right)=\left[\left(W1--W2\right)/W1\right]\times 100 \end{array}$$

Myoglobin (Mb) content was determined using the national standard method, as follows: weigh approximately 0.05 g of muscle tissue, add 1 mL of 40 mM PBS (pH 6.8), homogenize on an ice bath, and then centrifuge at 5,000 g for 10 min at 4°C. Filter the supernatant through filter paper, and measure the absorbance of the filtrate at 525, 545, 565, and 572 nm, recording the values as A1, A2, A3, and A4, respectively.


Mbconcentration(μmol/g)=(0.198×A1+0.176×A2+0.172×A3−0.332×A4×Vtotal÷W)

V total: 1 mL; W: 0.05 g.

### 2.4 Assessment of muscle antioxidant enzyme activity

The activities of antioxidant enzymes were measured using commercial kits (Beijing Leadman Biochemical Co., Ltd., Beijing, China). Total superoxide dismutase (T-SOD) and Malondialdehyde (MDA) were detected by the colorimetric method (Cat. No.: SOD-1-W, MDA-1-Y); Catalase (CAT) was measured by the ultraviolet absorption method (Cat. No.: CAT-1-Y); Glutathione peroxidase (GSH-Px) was determined by the enzymatic method (Cat. No.: GPX-1-W). All assays were performed using a microplate reader (Bio-Tek ELX800) in strict accordance with the manufacturer's instructions.

### 2.5 Sugar degradation index and metabolic enzyme activities assay

The sugar degradation index and metabolic enzyme activities assay were determined using commercially available kits from Beijing Leadman Biochemical Co., Ltd., Beijing, China. Muscle glycogen (Gly), lactate (LA), and glucose (Glu) were measured using the anthrone colorimetric method, the WST-8 colorimetric method, and the GOD-POD colorimetric method, respectively. The activities of succinic dehydrogenase (SDH) and lactate dehydrogenase (LDH) were determined using the 2,6-dichloroindophenol colorimetric and the 2,4-dinitrophenylhydrazine colorimetric method, respectively. NAD-dependent malate dehydrogenase (NAD-MDH), hexose kinase (HK) and pyruvate kinase (PK) were measured using ultraviolet absorption spectroscopy. The counts of Gly, LA, Glu, SDH, LDH, NAD-MDH, HK and PK were measured using commercial kits (Cat. No.: TY-1-Y, LA-1-G, PT-1-Y, SDH-1-Y, LDH-1-Y, NMDH-1-Y, HK-1-Y and PK-1-Y, respectively).

### 2.6 Mitochondrial respiratory chain enzyme activity

The activities of mitochondrial respiratory chain complex I (MRCC I), mitochondrial respiratory chain complex II (MRCC II), mitochondrial respiratory chain complex III (MRCC III), and mitochondrial respiratory chain complex IV (MRCC IV) were measured using commercial kits (Cat. No.: FHTA-1-Y, FHTB-1-Y, FHTC-1-Y, and FHTD-1-Y, respectively). All ELISA kits were purchased from Beijing Leadman Biochemical Co., Ltd., Beijing, China and all procedures were performed strictly in accordance with the manufacturer's instructions.

### 2.7 NADH-TR muscle fiber typing staining

After thawing the frozen sections at room temperature for 20 min, soak them in pure water for 5~10 min to remove the OCT embedding medium. Using a tissue brush, draw a circle around the tissue, then place the section in a wet box. Drop the NADH-TR staining solution in the circle to completely cover the tissue. Place it horizontally in a 37°C constant temperature incubator and incubate for 30 min under light protection, adding a small amount of pure water at the bottom of the wet box to maintain humidity. Then, pour off the staining solution and rinse with distilled water for 1 min. Dehydrate the sections in 90, 95%, and anhydrous ethanol for 3 min each, followed by 5 min in xylene to clarify, and mount with neutral resin.

The prepared tissue sections were scanned using a PANNORAMIC panoramic scanner, and the fluorescent sections of muscle tissue were imaged at 200 × magnification using CaseViewer 2.4 software. Six fields of view were randomly selected from each section, and the numbers of fast-twitch and slow-twitch muscle fibers were counted separately, and the percentages of fast-twitch and slow-twitch muscle fibers were calculated.

### 2.8 Real-time quantitative PCR (RT-qPCR)

Total RNA was extracted from LD muscle using the RNA Extraction Kit (TianGen, Beijing, China), and the concentration of RNA samples was detected by an ultramicro spectrophotometer (Thermo Nano drop-2000). Reverse transcription kits (HiScript II Q RT SuperMix) for qPCR; The cDNA was synthesized in Nanjing, China. The relative quantification was performed using a real-time fluorescence quantitative PCR instrument. Reaction conditions: 40 cycles of 95°C for 10 s and 60°C for 30 s. β*-actin* was used as the internal reference gene for correction, and the data were analyzed by the method of 2^−Δ*ΔCT*^. Primer 5.0 software was used to design gene primers, and the primer information is shown in Table 2.

**Table 2:** Primer sequences used in this study.

Items	5′3′	Length/bp	Tm/°C	**Gene sequence no**.
SOD1	F: GATCATGGGTTCCACGTCCA	138	60 °C	NM_001145185.2
	R: CACATTGCCCAGGTCTCCAA			
SOD2	F: TCAATAAGGAGCAGGGACGC	156	60 °C	NM_001280703.1
	R: TGGCCTTCAGATAATCGGGC			
CAT	F:CCAGATGGACACAGGCACAT	160	60 °C	XM_060400055.1
	R: CAGGATCTTCGTGGGCAAGT			
GPX1	F: AACGTAGCATCGCTCTGAGG	123	60 °C	XM_004018462.5
	R: CTGATGCCCAAACTGGTTGC			
Keap1	F: CACCCATGAACACCATCCGA	132	60 °C	XM_027969637.3
	R: TCCACGTTTCTGTCTCCACG			
Nrf2	F:CTACGGGCAAAAGCTCTCCA	171	60 °C	XM_015093345.4
	R:TCTGCAATTCTGAGCAGCCA			
IGF-1	F:AGCAGGTGAAGATGCCAGTC	127	60 °C	NM_001009774.3
	R:AGAGCATCCACCAACTCAGC			
mToR	F:GCAGCAACAGCGAGAGTGAGG	137	60 °C	XM_027975551.2
	R:GGAACGGAAGAAGCCTTGGACAG			
FoxO3	F:AGAAGTTCCCCAGCGACTTG	159	60 °C	NM_001267889.1
	R:TCCCCACGTTCAAACCAACA			
MAFbx	F:CCGACAAAGGACAGCTGGAT	133	60 °C	XM_004011657.5
	R:TGATCGGTGCCCTTCCA			
MyHC I	F:CTTCTCCCTGATCCACTACGC	129	60 °C	XM_004010325.4
	R:CTGCTGAGCATCTTGAGGGAA			
MyHC IIa	F:ATGGTGGAGAGGAGGGAGTC	111	60 °C	XM_027974884.3
	R:CAGCAGGGGCTTGATCTTGA			
MyHC IIx	F:ACTATGCGGGAACAGTGGAC	137	60 °C	XM_004012706.5
	R:AGATGCTGCGCCAGAGAACA			
MyHC IIb	F:TGTCTCCAAAGCCAAGGGAAA	145	60 °C	XM_060395436.1
	R:ACCTGACTCTGTGTGTAGACG			
Cytc	F:TCTCTGACTATCGTCGCCCT	179	60 °C	XM_004014896.5
	R:CAACACATCGGCAGAAGCAC			
NRF1	F:TGCTGTGGCAACAGGAAAGA	176	60 °C	XM_060414825.1
	R:GGTTGGGTTTGGAGGGAGAG			
TFAM	F:TGGCACATCACAGGTAAAGC	164	60 °C	XM_027962472.2
	R:GGTCTTCTCGTCCAACTTCCA			
TFB1M	F:CGTGAAGCAGCTATCCCAGAA	161	60 °C	XM_012183173.4
	R:CCAGAAGTTCAGCAACACCG			
CS	F:CGCTTGTACCTCACCATCCA	157	60 °C	XM_004006584.6
	R:CTTCCTGATTCGCCAGTCCA			
ATP5MC1	F:CCAGACCAGTGTTGTCTCCC	146	60 °C	NM_001009396.1
	R:GAGACGGGTTCCTGGCATAG			
β-actin	F:AAGATCAAGATCATCGCGCC	120	60 °C	NM_001009784.3
	R:GGACTCGTCGTACTCCTGCT			

### 2.9 Metabolomics analysis

For metabolomics analysis, muscle samples from six slaughtered sheep per group (18 samples in total) were subjected to liquid chromatography-mass spectrometry (LC-MS). Metabolites were extracted by mixing 100 μL of thawed samples with 500 μL of extraction solvent (methanol–acetonitrile, 1:1, v/v) containing 2-chloro-L-phenylalanine (20 mg/L) as the internal standard. The mixture was vortexed for 30 s, subjected to ultrasonication for 10 min in an ice-water bath, and incubated at −20°C for 1 h. Subsequently, the sample was centrifuged at 12,000 rpm for 15 min at 4°C. A total of 500 μL supernatant was collected and concentrated to dryness, then reconstituted with 160 μL acetonitrile–water (1:1, v/v). The entire extraction procedure was repeated twice.

Tandem extraction was performed on a Waters Xevo Acquity I Class PLUS ultra-high performance liquid chromatograph (UPLC) and a Waters Xevo G2-XS QTOF High-resolution Mass Spectrometer (HRMS). A Waters Xevo Acquity UPLC HSS T3 column (1.8 um; 2.1^*^100 mm) was used. Progenesis QI software was used for peak extraction and peak comparison when analyzing metabolome data, and metabolites were identified based on the METLIN database and public databases. The theoretical fragment identification deviation and mass deviation were within 100 ppm. The repeatability of within-group results and quantity control sample results was determined using principal component analysis (PCA) and Spearman's correlation analysis. Finally, the differential metabolites were screened by combining the differential multiples in the OPLS-DA model, *t*-test *P* value and VIP value, and the screening standard was *P* value < 0.05 and VIP > 1. Moreover, KEGG pathway enrichment analysis was performed for differential metabolites (19).

### 2.10 Proteomics analysis

#### 2.10.1 Preparation and protein digestion

Muscle samples from six slaughtered sheep per group (18 samples in total) were subjected to proteomic analysis using the Astral DIA method. RIPA lysis buffer was added to the samples followed by homogenization, and ultrasonic crushing was performed in an ice bath. All samples were centrifuged at 4°C and 10,000 × g for 10 min, after which the supernatant was transferred into pre-chilled 1.5 mL EP tubes. DL-dithiothreitol (DTT) solution was supplemented to a final concentration of 10 mmol/L, and the mixture was incubated in a 56°C water bath for 1 h for reduction. Thereafter, iodoacetamide (IAM) solution was added to reach a final concentration of 55 mmol/L, and the sample was incubated for 40 min under light-protected conditions. Centrifuge to discard the supernatant and add 50 mmol/L NH_4_HCO_3_ to 100 μL. Pre-chilled acetone was then added to the sample, and the mixture was frozen overnight at −20°C to form protein pellets. The samples were centrifuged again at 10,000 × g for 10 min at 4°C; the EP tubes were carefully inverted to remove acetone. Subsequently, the enzyme was added to the protein solution, and the mixture was incubated overnight at 37°C for enzymatic digestion. After digestion, the peptides were desalted using a self-priming desalting column, and the solvent was removed by vacuum centrifuge at 45°C. Finally, the peptides were dissolved in the sample solution (0.1% formic acid), fully vortexed, and centrifuged at 13,200 rpm for 10 min at 4°C. The resulting supernatant was transferred to a sample tube for subsequent mass spectrometry analysis.

#### 2.10.2 Nano LC-MS/MS analysis

Nano LC-MS/MS analysis was conducted on a Vanquish (ThermoFisher Scientific, USA) UHPLC system coupled to an Orbitrap Astral Mass Spectrometer (Thermo Fisher Scientific, USA). Peptide separation was performed on a 100 μm i.d. × 180 mm column packed with 3 μm Reprosil-Pur 120 C18-AQ media. Mobile phase A was 0.1% aqueous formic acid, and mobile phase B consisted of 0.1% formic acid in water/acetonitrile (20:80), with a flow rate of 600 nL/min. MS acquisition adopted MasterScan mode at a resolution of 240,000 with a scan range of 380–980 m/z. The raw MS files were analyzed and searched against a target protein database based on the species of the samples using MaxQuant (1.6.2.10). Differentially expressed proteins (DEPs) were screened for FC ≥ 1.2, *P* < 0.05. Then, KEGG and Pfam enrichment analysis for the DEPs was performed using the clusterProfiler software.

### 2.11 Statistical analyses

Data on the meat quality, muscle fiber traits, antioxidant capacity and mitochondrial function were analyzed using a one-way ANOVA analysis procedure with Duncan's test. SPSS (version 25.0, Chicago, IL, USA) was used for all statistical procedures. Statistical results are shown as mean and standard error, and *P* < 0.05 was considered statistically significant. Spearman correlation test was used to calculate the correlation between differential muscle fiber traits, antioxidant capacity and mitochondrial function, differential metabolites and DEPs. The range of the correlation coefficient (*r*) was from −1 to 1. *r* > 0 and < 0 represented positive correlation and negative correlation, respectively. The |*r*| value denoted the correlation degree among variables.

## 3 Results

### 3.1 Meat quality and muscle antioxidant capacity

**Figure 1:**
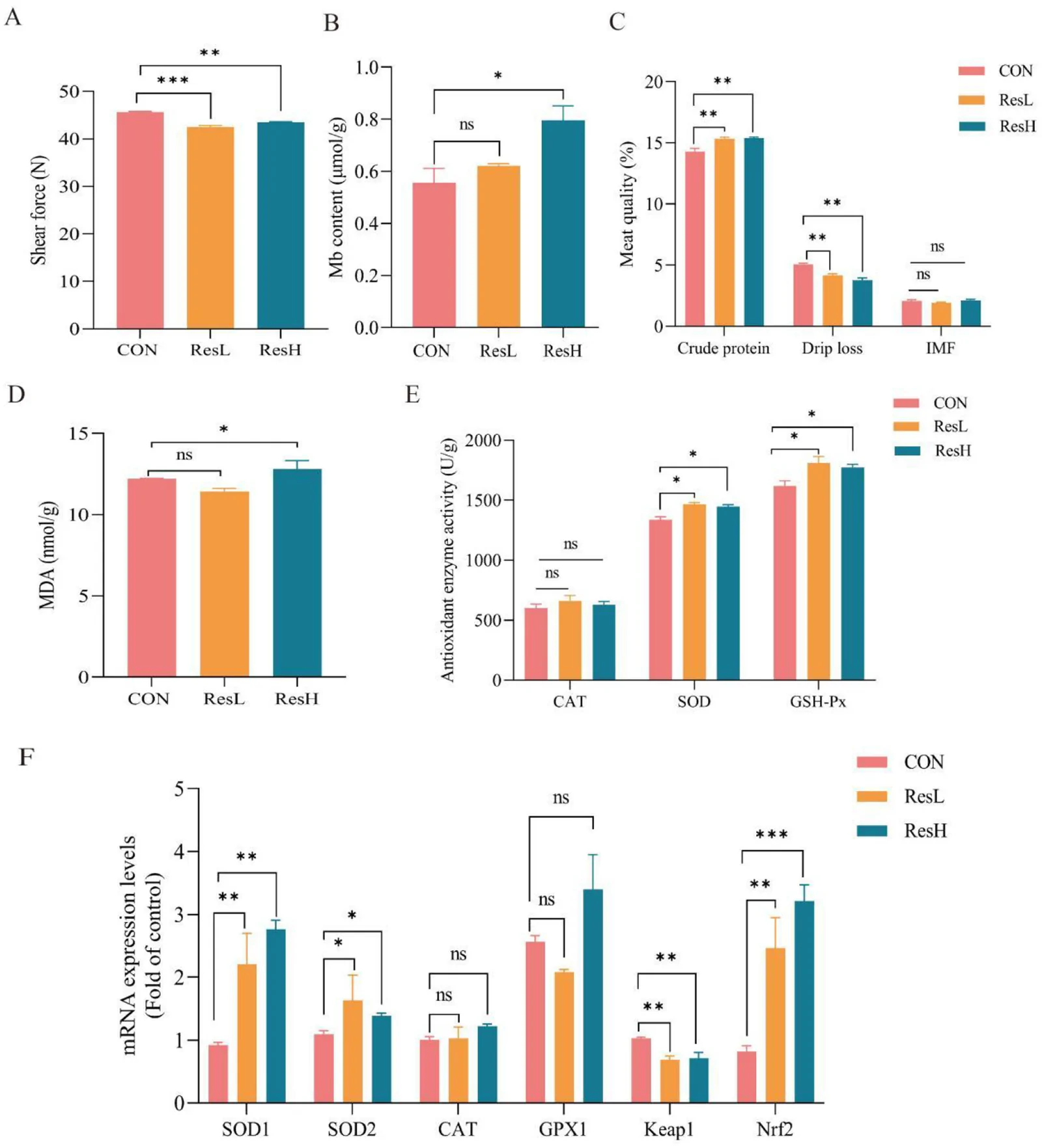
Effect of dietary RES supplementation on meat quality and antioxidant capacity of Tibetan sheep (*n* = 6). Mb = myoglobin protein; MDA = malonaldehyde; CAT = catalase; SOD = total superoxide dismutase; GSH-Px = glutathione peroxidase; *SOD1* = superoxide dismutase 1; *SOD2* = superoxide dismutase 2; *CAT* = catalase; *GPX1* = glutathione peroxidase 1; Nrf2 = nuclear factor erythroid 2-related factor 2; *Keap1* = Kelch-like ECH-associated protein 1; CON = control; ResL = basal diet + 80 mg/kg RES; ResH = basal diet + 360 mg/kg RES. *P < 0.05, **P < 0.01, ***P < 0.001.

Dietary RES supplementation improved meat quality and muscle antioxidant capacity in Tibetan sheep. Compared with the CON group, both ResH and ResL groups showed significantly decreased shear force and drip loss, along with increased crude protein content (*P* < 0.05). Additionally, myoglobin (Mb) content was significantly elevated in the ResH group (*P* < 0.05; Figures 1A–C). Regarding muscle antioxidant capacity, SOD and GSH-Px activities were significantly increased in both treatment groups (*P* < 0.05; Figure 1E), while MDA level was reduced in the ResH group (*P* < 0.05; Figure 1D). Furthermore, the mRNA expression of *SOD1, SOD2*, and *Nrf2* was significantly upregulated in both treatment groups (*P* < 0.05), while *Keap1* expression was significantly downregulated (*P* < 0.01; Figure 1F).

### 3.2 Muscle fiber type transformation and skeletal muscle development

**Figure 2:**
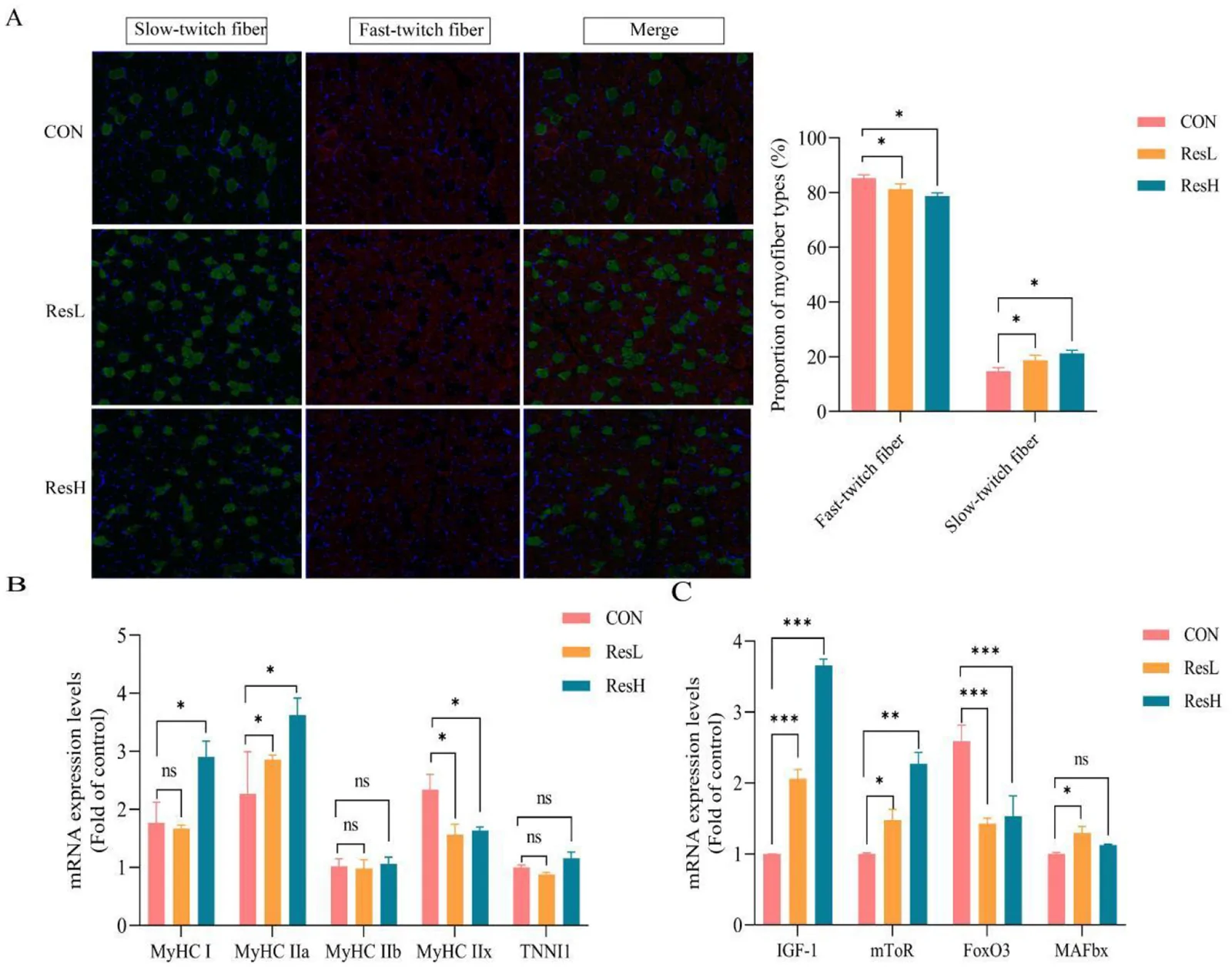
Effect of dietary RES supplementation on muscle fiber type transformation and skeletal muscle development of Tibetan sheep (*n* = 6). **(A)** NADH-TR muscle fiber typing staining analyzed the percentage of slow-twitch fiber and fast-twitch fiber. All photographs were captured at 200x magnification. **(B)** Real-time quantitative PCR analyzed the mRNA levels of *MyHC I, MyHC IIa, MyHC IIx, MyHC IIb* and troponin I type 1 (*TNNI1*). **(C)** The mRNA levels of skeletal muscle development-related genes including insulin-like growth factor 1 (*IGF-1*), mechanistic target of rapamycin (*mTOR*), forkhead box O3 (*FoxO3*), and Muscle Atrophy F-box Protein (*MAFbx*). CON = control; ResL = basal diet + 80 mg/kg RES; ResH = basal diet + 360 mg/kg RES *P < 0.05, **P < 0.01, ***P < 0.001.

Compared with the CON group, dietary RES supplementation increased the proportion of slow-twitch fibers and decreased the proportion of fast-twitch fibers (*P* < 0.05; Figure 2A). Meanwhile, the mRNA expression of *MyHC IIa* was significantly upregulated, whereas *MyHC IIx* was downregulated in both treatment groups (*P* < 0.05; Figure 2B). Furthermore, dietary RES supplementation upregulated the expression of genes associated with skeletal muscle development (including *IGF-1* and *mTOR*), while downregulating *FoxO3* expression (*P* < 0.05; Figure 2C).

### 3.3 Mitochondrial function and metabolic enzyme activity

**Figure 3:**
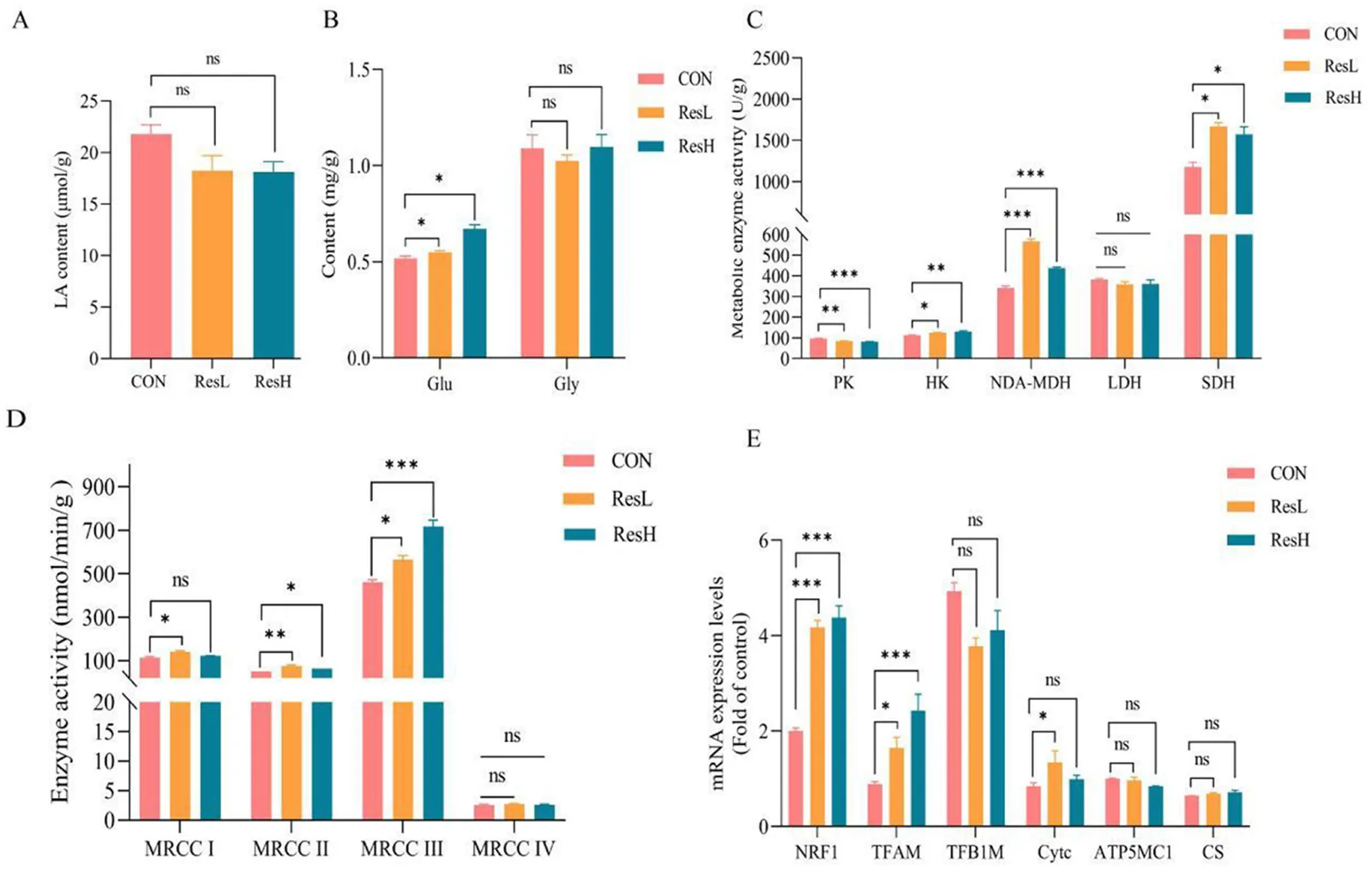
Effect of dietary RES supplementation on mitochondrial function and metabolic enzyme activity of Tibetan sheep (*n* = 6). **(A)** Lactate (LA) content. **(B)** Glycogen (Gly) and glucose (Glu) contents. **(C)** The activities of pyruvate kinase (PK), hexokinase (HK), succinic dehydrogenase (SDH), NAD-dependent malate dehydrogenase (NAD-MDH) and lactate dehydrogenase (LDH) enzymes. **(D)** The activities of mitochondrial respiratory chain complexes including MRCC I, MRCC II, MRCC III, and MRCC IV. **(E)** The mRNA levels of mitochondrial biogenesis-related genes including nuclear respiratory factor 1 (*NRF1*), mitochondrial transcription factor A (*TFAM*), mitochondrial transcription factor B1 (*TFB1M*), cytochrome c (*Cytc*), ATP synthase membrane subunit c locus 1 (*ATP5MC1)* and citrate synthase (CS). CON = control; ResL = basal diet + 80 mg/kg RES; ResH = basal diet + 360 mg/kg RES. *P < 0.05, **P < 0.01, ***P < 0.001.

Dietary RES supplementation had no significant effect on muscle lactate (LA) and glycogen (Gly) contents (*P* > 0.05), but significantly increased glucose (Glu) content (*P* < 0.05; Figures 3A, B). Regarding metabolic enzymes and mitochondrial function, RES reduced pyruvate kinase (PK) activity while significantly enhancing the activities of hexokinase (HK), succinate dehydrogenase (SDH), and NAD-dependent malate dehydrogenase (NAD-MDH), as well as mitochondrial respiratory chain complexes II and III (MRCC II and MRCC III) in the muscle (*P* < 0.05; Figures 3C, D). Meanwhile, the expression of mitochondrial function-related genes, including *NRF1* and *TFAM*, was significantly upregulated in both treatment groups (*P* < 0.05; Figure 3E).

### 3.4 Metabolic spectrum analysis of the LD muscle

The metabolome of 18 muscle samples was qualitatively and quantitatively analyzed by the LC-MS platform. In total, 2,708 metabolites (positive and negative ion mode) were detected. PCA clustering analysis revealed clear separation of the metabolic profiles in both ResH and ResL groups from those of the CON group (Supplementary Figures S1A, B). Permutation testing showed that the model between metabolites and experimental treatment is stable and reliable, and the predictive effect is good (Supplementary Figures S1C, D). Then, analysis with FC > 1.2, *P* value < 0.05, and VIP > 1 as screening criteria revealed that 243 and 428 differential metabolites were identified between the CON vs. ResL and CON vs. ResH groups, respectively (Supplementary Figures S1E, F).

**Figure 4:**
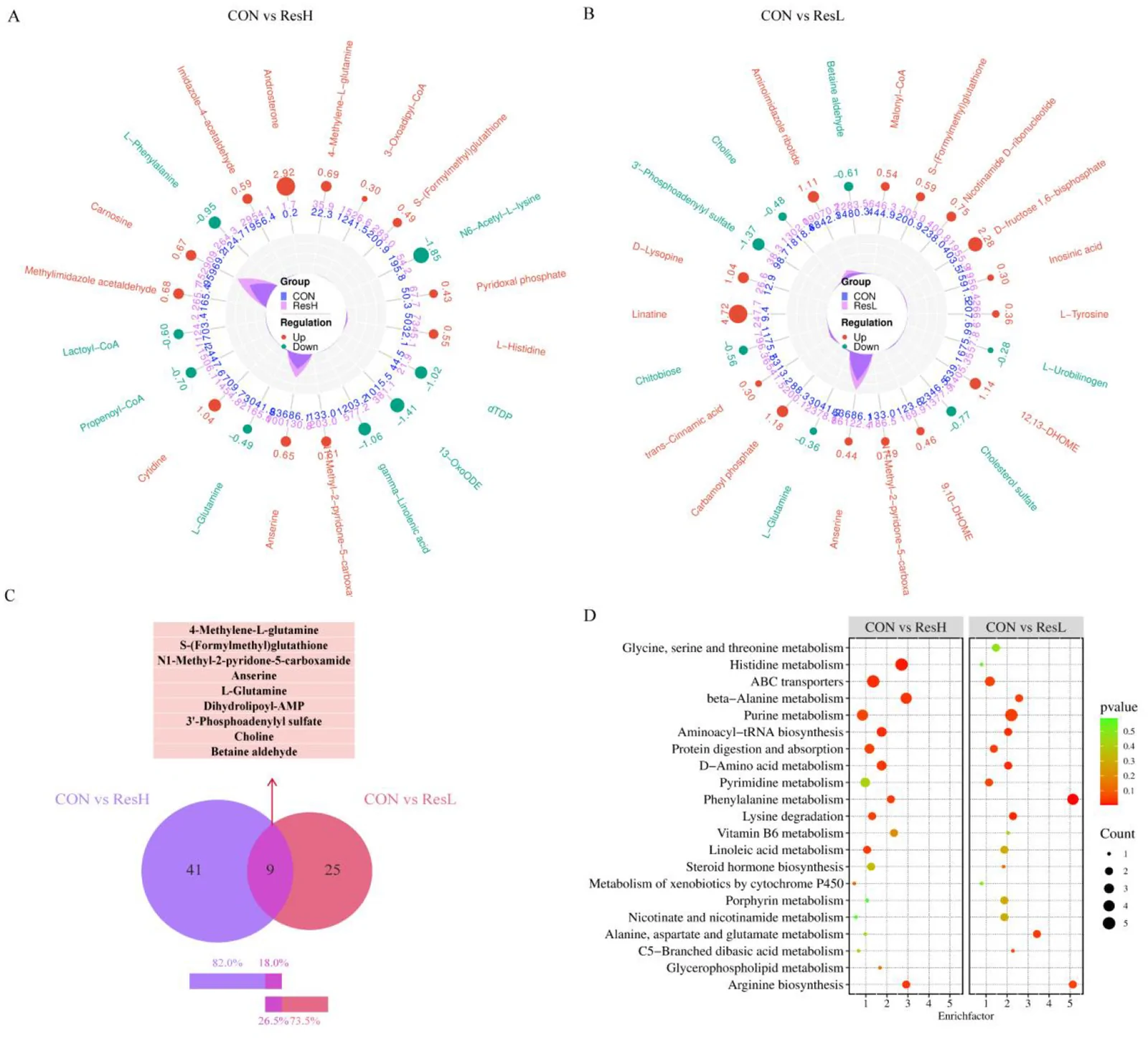
Effect of dietary RES supplementation on muscle metabolite profile of Tibetan sheep (*n* = 6). **(A)** Radar chart of key metabolites in the ResH group. **(B)** Radar chart of key metabolites in the ResL group. **(C)** Venn diagram of shared metabolites between CON vs. ResL and CON vs. ResH. **(D)** The KEGG functional enrichment map of differential metabolites.

Through screening, 51 key metabolites associated with skeletal muscle energy supply, muscle fiber growth, meat quality, and oxidative homeostasis balance were identified in the ResH group (Supplementary Table S1). Among these, 3-oxoadipyl-CoA, pyridoxal phosphate, L-histidine, anserine, cytidine, methylimidazole acetaldehyde, carnosine, imidazole-4-acetaldehyde, and androsterone were significantly upregulated, while N6-acetyl-L-lysine, dTDP, 13-oxoODE, γ-linolenic acid, L-glutamine, propenoyl-CoA, and lactoyl-CoA were significantly downregulated (*P* < 0.05; Figure 4A). In the ResL group, 35 metabolites were identified as significantly associated with the aforementioned functions (Supplementary Table S2). Among them, malonyl-CoA, nicotinamide D-ribonucleotide, D-fructose-1,6-bisphosphate, inosinic acid, L-tyrosine, anserine, carbamoyl phosphate, trans-cinnamic acid, linatine, and D-lysopine were significantly upregulated, while L-urobilinogen, cholesterol sulfate, L-glutamine, chitobiose, choline, and betaine aldehyde were significantly downregulated (*P* < 0.05; Figure 4B).

Of particular significance, this study identified nine metabolites co-regulated across both treatment groups (Figure 4C). Of these, 4-methylene-L-glutamine, S-(formylmethyl)glutathione, N1-methyl-2-pyridone-5-carboxamide, and anserine were upregulated, whereas L-glutamine, dihydrolipoyl-AMP,3′-phosphoadenylyl sulfate, choline, and betaine aldehyde were downregulated. KEGG functional enrichment analysis revealed that the key metabolites in both treatment groups were collectively enriched in amino acid metabolism-related pathways, ABC transporters, Vitamin B6 metabolism, linoleic acid metabolism, steroid hormone biosynthesis, nicotinate and nicotinamide metabolism pathway (Figure 4D).

### 3.5 DIA-based quantification of key proteins

**Figure 5:**
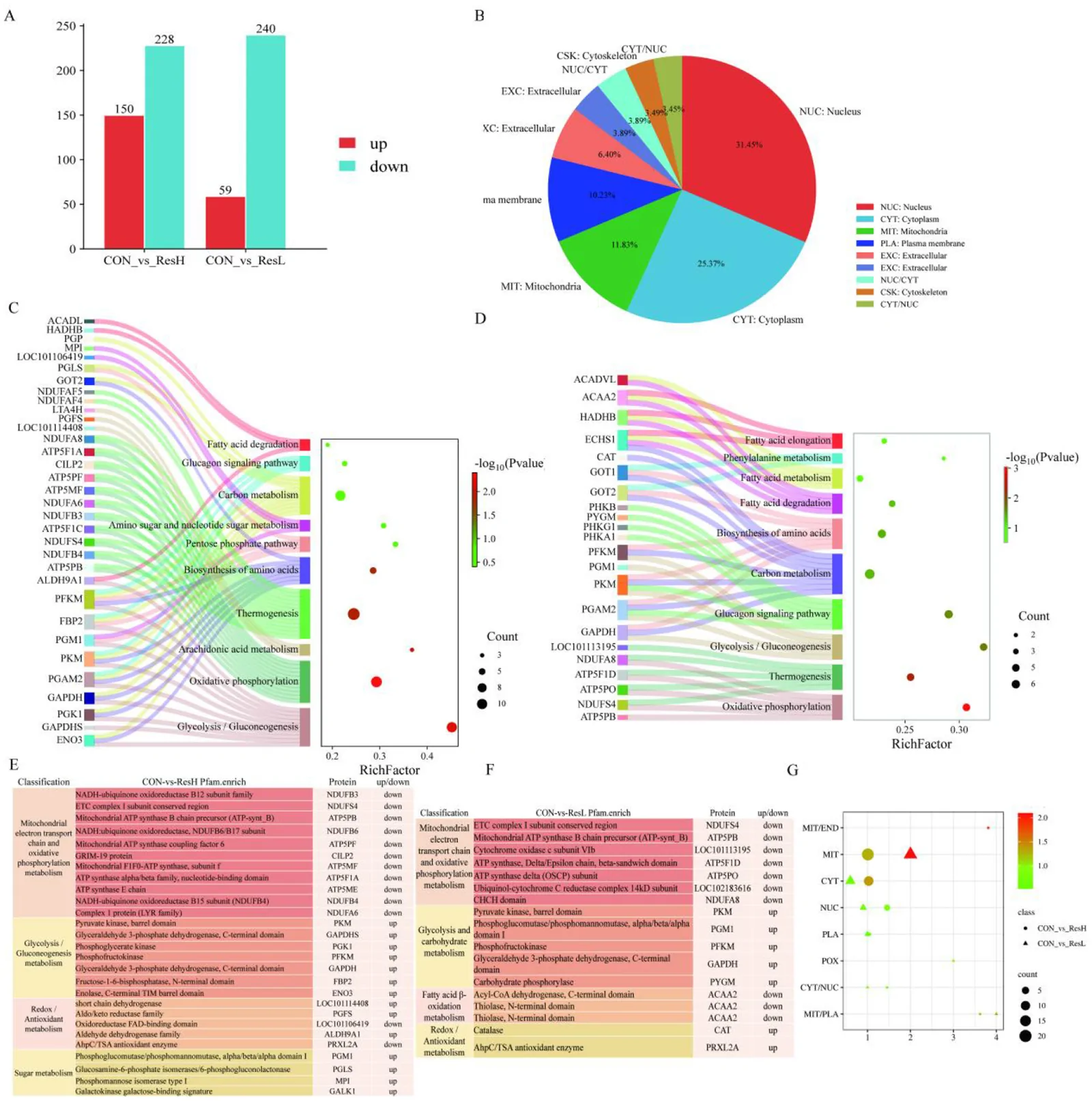
DIA-based quantification and enrichment analysis of key proteins. **(A)** DEP counts. **(B)** Subcellular localization analysis. **(C)** DEPs enriched in related pathway in CON vs. ResH. **(D)** DEPs enriched in the related pathway in CON vs. ResL. **(E)** Pfam enrichment analysis between CON and ResH. **(F)** Pfam enrichment analysis between CON and ResL. **(G)** Subcellular localization analysis of key proteins. All values were expressed as means ± SEM, *n* = 6. CON: control; ResL: basal diet + 80 mg/kg RES; ResH: basal diet + 360 mg/kg RES.

In this study, data-independent acquisition (DIA) was employed to profile the muscle proteome, and 2,259 proteins were quantified. PCA results showed distinct separation between CON vs. ResL and CON vs. ResH (Supplementary Figures S2A, B), indicating that dietary RES supplementation altered muscle protein expression in Tibetan sheep. Among all the identified proteins, analysis with FC > 1.2, *P* value < 0.05, and VIP > 1 as screening criteria revealed that 299 and 378 DEPs were identified between the CON vs. ResL and CON vs. ResH groups, respectively (Supplementary Figures S2C, D; Figure 5A). The subcellular localization analysis indicated that DEPs were predominantly located in the nucleus (31.45%) and cytoplasm (25.37%; Figure 5B).

Through screening, 39 key proteins associated with mitochondrial oxidative phosphorylation, glycolysis/glucose gluconeogenesis, fatty acid β-oxidation, antioxidant stress, and amino acid metabolism were identified in the ResH group (Supplementary Table S3). In the ResL group, 28 key proteins were identified as significantly associated with the aforementioned functions (Supplementary Table S4). KEGG functional enrichment analysis revealed that the key proteins in both treatment groups were collectively enriched in fatty acid degradation, glucagon signaling pathway, carbon metabolism, biosynthesis of amino acids, thermogenesis, glycolysis/gluconeogenesis and oxidative phosphorylation pathway (Figures 5C, D).

Further, Pfam enrichment analysis revealed that the key proteins were collectively enriched in pathways related to the mitochondrial electron transport chain, oxidative phosphorylation, glycolysis/gluconeogenesis and redox/antioxidant metabolism (Figures 5E, F). Compared with the CON group, the downregulated proteins primarily included NDUFB3, NDUFS4, ATP5PB, NDUFB6, ATP5PF, CILP2, ATP5MF, and NDUFB4 in the ResH group, while the upregulated proteins mainly consisted of PKM, GAPDHS, PGK1, PFKM, GAPDH, FBP2, ENO3, ALDH9A1, PRXL2A, PGM1, PGLS, MPI and GALK1 (Figure 5E). In the ResL group, the downregulated proteins primarily included NDUFS4, ATP5PB, ATP5F1D, ATP5PO, NDUFA8, while the upregulated proteins mainly consisted of PKM, PGM1, PFKM, GAPDH, PYGM, ACAA2, CAT and PRXL2A (Figure 5F). Importantly, we found that HADHB, NDUFS4, GOT2 and ATP5PB were significantly downregulated in both treatment groups, while PKM, PGM1, PFKM, PGAM2 and GAPDH were significantly upregulated. Finally, subcellular localization studies of these proteins revealed their predominant distribution in the cytoplasm and mitochondria (Figure 5G).

### 3.6 Correlation analysis

**Figure 6:**
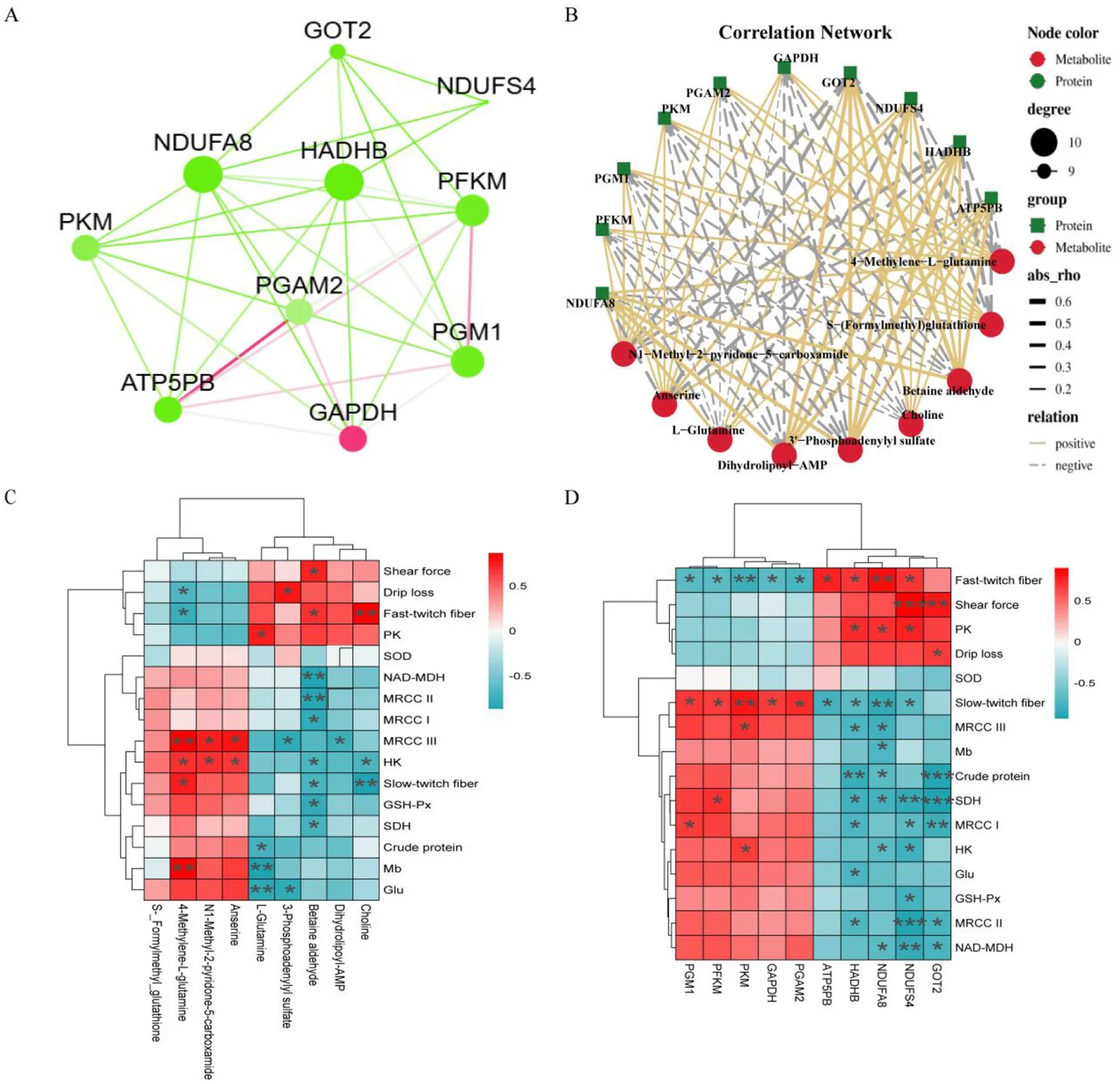
Correlation analysis. **(A)** Key protein interaction network diagram. **(B)** Correlation between co-regulated metabolites and proteins in both treatment groups. **(C)** Correlations between key metabolites and muscle quality, antioxidant capacity, and muscle fibers. **(D)** Correlations between key proteins and muscle quality, antioxidant capacity, and muscle fibers. * *p* < 0.05, ** *p* < 0.01, *** *p* < 0.001

This study conducted correlation network analysis on the identified key differential proteins. NDUFA8, HADHB, and PFKM served as core hub proteins in the network, exhibiting significant positive correlations with most other proteins. Notably, GAPDH showed significant negative correlations with ATP5PB, PGAM2 and PGM1 (*P* < 0.05; Figure 6A). The metabolite-protein correlation network revealed that key glycolytic enzymes (PFKM, PKM, PGAM2, PGM1, and GAPDH) were positively correlated with 4-methylene-L-glutamine, anserine, N1-methyl-2-pyridone-5-carboxamide, and S-(formylmethyl)glutathione, but negatively correlated with L-glutamine, dihydrolipoyl-AMP,3′-phosphoadenylyl sulfate, choline, and betaine aldehyde. In contrast, mitochondrial respiratory chain proteins (NDUFA8, NDUFS4, and ATP5PB) were consistently negatively correlated with L-glutamine, choline, and3′-phosphoadenylyl sulfate (*P* < 0.05; Figure 6B).

Correlation analysis between metabolites and meat quality/physiological traits revealed that the upregulated metabolites 4-methylene-L-glutamine, N1-methyl-2-pyridone-5-carboxamide, and anserine were positively correlated with HK and MRCC III (*P* < 0.05; Figure 6C), the downregulated metabolite betaine aldehyde was negatively correlated with NAD-MDH, HK, SDH, slow-twitch fiber, and GSH-Px, while being positively correlated with shear force and fast-twitch fiber (*P* < 0.05). Additionally, L-glutamine was negatively correlated with crude protein, Mb and Glu (*P* < 0.05; Figure 6C). Further analysis revealed that fast-twitch fiber and slow-twitch fiber exhibited significant correlations with all key proteins (*P* < 0.05). The HADHB, NDUFA8, NDUFS4, and GOT2 showed significant correlations with muscle antioxidant enzymes, metabolic enzymes, mitochondrial respiratory chain, meat quality, and muscle fiber characteristics (*P* < 0.05; Figure 6D).

## 4 Discussion

RES is a natural polyphenol that has been reported to enhance muscle antioxidant capacity, muscle fiber characteristics and mitochondrial biogenesis in animals (14, 20–22). Our results further indicated that dietary RES supplementation effectively improves meat quality, antioxidant capacity, muscle fiber type transformation, skeletal muscle development and mitochondrial biogenesis of Tibetan sheep.

Meat quality is closely related to muscle antioxidant capacity, and dietary antioxidant supplementation has been shown to effectively improve meat quality (23, 24). In this study, dietary RES supplementation at 80 or 360 mg/kg significantly reduced muscle shear force and drip loss, while increasing crude protein content of Tibetan sheep. Shear force is a direct indicator of meat tenderness, and drip loss reflects water-holding capacity; both are key parameters for evaluating the edible quality of mutton (25). The reduction in shear force and drip loss observed in this study suggests that RES improved the sensory and processing qualities of Tibetan sheep muscle, which is consistent with previous findings in pork (14) and beef (26). The increase in crude protein content may be attributed to RES-induced promotion of muscle protein synthesis or inhibition of protein degradation, as supported by the upregulation of *IGF-1* and *mTOR* and the downregulation of *FoxO3* observed in our study, in line with earlier reports that RES activates the Akt/mTOR pathway (27) and suppresses FoxO-mediated proteolysis (28).

The meat quality improvement induced by RES may be achieved by enhancing muscle antioxidant capacity. The Nrf2/Keap1 signaling pathway is a key pathway that regulates the antioxidant genes expression. Under resting conditions, Keap1 binds to Nrf2 and promotes its degradation; upon stimulation by bioactive compounds such as RES, Nrf2 dissociates from Keap1 and translocates into the nucleus (29), thereby initiating the transcription of downstream antioxidant genes (e.g., *SOD1, SOD2*, and *GSH-Px*) (30). In agreement with this mechanism, our study showed that RES supplementation upregulated the expression of *Nrf2* and its target genes *SOD1* and *SOD2*, while downregulating *Keap1* expression, accompanied by increased activities of SOD and GSH-Px. SOD serves as the first line of defense against superoxide anions, and GSH-Px catalyzes the reduction of hydrogen peroxide and organic peroxides; together, they constitute a crucial enzymatic antioxidant system (31, 32). This indicates that RES enhances the antioxidant defense system in Tibetan sheep muscle by inhibiting Keap1 expression and activating Nrf2. Notably, this study found that a high dose (360 mg/kg) of RES further reduced MDA content and increased myoglobin content, suggesting that a high dosage could more effectively alleviate muscle oxidative damage and stabilize meat color. Collectively, these differences preliminarily indicated that the higher RES dose (360 mg/kg) was more effective in improving muscle antioxidant status and meat color stability.

Muscle fiber type composition is an intrinsic determinant of meat quality. In this study, RES supplementation increased the proportion of slow-twitch fibers, decreased fast-twitch fibers, upregulated *MyHC IIa* expression, and downregulated *MyHC IIx* expression. Slow-twitch fibers are rich in mitochondria and myoglobin and rely predominantly on oxidative metabolism, which is closely associated with high tenderness, stable meat color, and good water-holding capacity (33). The finding that RES induces a shift in muscle fiber type toward the oxidative type is consistent with the findings of Zhang et al. (14) in porcine muscle, which supports the improvements in meat quality observed in this study (i.e., increased tenderness, reduced drip loss, and increased myoglobin content).

Metabolic enzyme activities and mitochondrial function indirectly indicate muscle fiber type composition. In this study, RES significantly reduced PK activity while elevating HK, SDH, and NAD-MDH activities, indicating a clear shift from glycolytic to aerobic oxidative metabolism. As the rate-limiting enzyme of glycolysis, decreased PK activity limits anaerobic glycolysis, thereby alleviating meat quality deterioration such as higher water loss and lower tenderness (12). In contrast, HK, SDH, and NAD-MDH are key enzymes in aerobic oxidative pathways. Elevated HK activity promotes glucose uptake and entry into aerobic metabolism (34), consistent with the increased muscle glucose content observed in this study. SDH, a marker enzyme of the tricarboxylic acid (TCA) cycle, and NAD-MDH, which participates in the TCA cycle-linked malate-aspartate shuttle, both exhibited enhanced activities, directly reflecting improved mitochondrial oxidative capacity and energy metabolic balance (35). The synergistic enhancement of these three enzyme activities elevates the aerobic metabolic capacity of skeletal muscle. Mitochondria, as the core site of aerobic metabolism, have functions closely associated with muscle fiber type and meat quality (36). In this study, RES supplementation increased the activities of MRCC II and MRCC III, which are key complexes involved in electron transport and ATP production. Their enhanced activities improve mitochondrial ATP synthesis efficiency, thereby providing adequate energy for muscle contraction, protein synthesis, and antioxidant defense (37). Concurrently, the mRNA expression of mitochondrial biogenesis-related genes *NRF1* and *TFAM* was significantly upregulated in both treatment groups. *NRF1* serves as a key transcription factor regulating *TFAM* expression, whereas *TFAM* is directly involved in mitochondrial DNA transcription and replication, playing a critical role in maintaining mitochondrial function (18). The upregulation of *NRF1* and *TFAM* thus indicates that RES promotes mitochondrial biogenesis, further reinforcing oxidative metabolism and providing sufficient energy support for oxidative slow-twitch fibers. Collectively, the increased activities of SDH and NAD-MDH, enhanced MRCC II/III function, and upregulated *NRF1/TFAM* expression demonstrate that RES improves muscle oxidative capacity through enhancing mitochondrial function.

To elucidate the molecular mechanisms underlying RES-mediated improvements in meat quality, antioxidant capacity, muscle fiber characteristics, and mitochondrial function in Tibetan sheep, we performed untargeted metabolomics and proteomics analyses. Metabolomic profiling identified 51 and 35 key metabolites in the ResH and ResL groups, respectively, which were associated with skeletal muscle energy supply, fiber growth, meat quality, and redox homeostasis. Among these, nine metabolites exhibited consistent alterations across both treatment groups and may represent primary targets of RES action. Specifically, 4-methylene-L-glutamine, S-(formylmethyl) glutathione, and anserine were upregulated, whereas L-glutamine, dihydrolipoyl-AMP,3′-phosphoadenylyl sulfate (PAPS), choline, and betaine aldehyde were downregulated. L-glutamine is the predominant free amino acid in skeletal muscle, serving as both an important carbon skeleton donor for the TCA cycle and a key precursor for glutathione synthesis (38). Its reduced levels indicate that RES promotes glutamine catabolism and utilization, thereby facilitating glutathione synthesis and reinforcing antioxidant defense. Anserine is known to scavenge free radicals and inhibit lipid peroxidation (39). Therefore, its upregulation in this study offers a reasonable explanation for the improved antioxidant capacity after RES supplementation. Meanwhile, choline and betaine aldehydes are key intermediates in methylation metabolism and phospholipid synthesis (40, 41); their downregulation indicates that RES can remodel muscle methylation metabolism and membrane phospholipid composition (42).

Additionally, we found that carnosine was significantly upregulated in the ResH group. Carnosine is structurally similar to anserine and exhibits antioxidant properties, which protect muscle proteins against oxidative damage (43). Its upregulation was accompanied by increased L-histidine, which provides the substrate for both carnosine and anserine synthesis, suggesting that RES promotes the biosynthesis of these bioactive dipeptides. The exclusive elevation of carnosine in the high-dose group further indicated that 360 mg/kg RES exerted a stronger regulatory effect on antioxidant dipeptide metabolism. Pathway enrichment analysis revealed that the differentially expressed metabolites in both groups were commonly enriched in the ABC transporter pathway and the aminoacyl-tRNA biosynthesis pathway. ABC transporters are involved in the transmembrane transport of amino acids, lipids, and other molecules, and play a role in reshaping the distribution of intracellular metabolites (44). Aminoacyl-tRNA biosynthesis is closely related to protein translation (45). Its enrichment is consistent with the activation of the IGF-1/mTOR pathway and increased muscle crude protein, indicating that RES promotes muscle protein deposition through metabolic substrates and translation efficiency.

Proteomics analysis revealed that 39 and 28 key proteins related to skeletal muscle energy supply, muscle fiber growth, muscle quality, and redox homeostasis were identified in the ResH and ResL groups, respectively. These proteins were primarily enriched in pathways involving fatty acid degradation, oxidative phosphorylation, glycolysis/glucogenesis, and amino acid biosynthesis, with most localized in the cytoplasm and mitochondria. Key glycolytic enzymes (PKM, PFKM, PGM1, PGAM2, and GAPDH) were significantly upregulated in both groups. Glycolysis is the central pathway of muscle energy metabolism, and the overall upregulation of these key enzymes indicates that RES remodels the muscle glycolytic metabolic pattern (46). Among these, PFKM and PKM catalyze the key irreversible reactions at the upstream and downstream of glycolysis, respectively, and serve as the core rate-limiting sites regulating glycolytic flux (47). Their simultaneous upregulation suggests that RES can holistically enhance muscle glycolytic metabolic potential. PGM1 mediates the reversible interconversion between glucose-6-phosphate and glucose-1-phosphate, serving as a critical hub linking glycolysis with glycogenesis and glycolysis (48, 49); its upregulation enables muscles to flexibly switch between glycogen storage and glycolytic energy supply modes according to energy demands. PGAM2 maintains the smooth flow of intermediate metabolites in glycolysis (50). In addition to its role in glycolysis, GAPDH also mediates oxidative stress responses (51). In this study, GAPDH was co-upregulated with antioxidant dipeptides including anserine and carnosine, suggesting that GAPDH may also contribute to RES-mediated antioxidant regulation through its non-glycolytic functions.

Notably, the mitochondrial respiratory chain subunits NDUFS4 and ATP5PB were significantly downregulated, whereas overall mitochondrial function was still enhanced, showing a pattern of lower protein abundance but higher functional activity. This seemingly paradoxical observation may be attributed to the activation of mitochondrial quality control (MQC) mechanisms, including mitophagy, fusion-fission dynamics, and cristae remodeling (52). Under conditions of RES stimulation, cells may enhance mitochondrial efficiency by eliminating dysfunctional organelles via mitophagy while simultaneously promoting mitochondrial biogenesis (53, 54). Additionally, the reorganization of respiratory complexes into supercomplexes (SCs) can optimize electron transfer efficiency without requiring increased expression of individual subunits (55). The downregulation of NDUFS4 may also reflect Complex I remodeling, as this subunit is essential for Complex I stability (56). Collectively, RES appears to improve mitochondrial function primarily through quality optimization and architectural remodeling rather than through simple upregulation of respiratory chain components. Concurrently, the fatty acid metabolism protein HADHB was downregulated, suggesting that RES may have reshaped the preference of muscle fatty acid oxidation pathways (57). Correlation analysis revealed that DEPs such as HADHB, NDUFA8, NDUFS4, and GOT2 were significantly associated with muscle antioxidant capacity, muscle fiber traits, metabolic enzyme activity, mitochondrial function, and meat quality, serving as central nodes integrating skeletal muscle energy metabolism, oxidative homeostasis, and meat quality regulation. Furthermore, upregulated metabolites (4-methylene-L-glutamine, N1-Methyl-2-pyridone-5-carboxamide, and anserine) showed positive correlations with HK and MRCC III; whereas downregulated metabolites (betaine aldehyde) showed negative correlations with NAD-MDH, HK, SDH, slow-twitch fibers, and GSH-Px, but positive correlations with shear force and fast-twitch fibers. These results indicate that RES influences meat quality, antioxidant capacity, and muscle fiber characteristics by modulating muscle proteins and metabolites.

**Figure 7:**
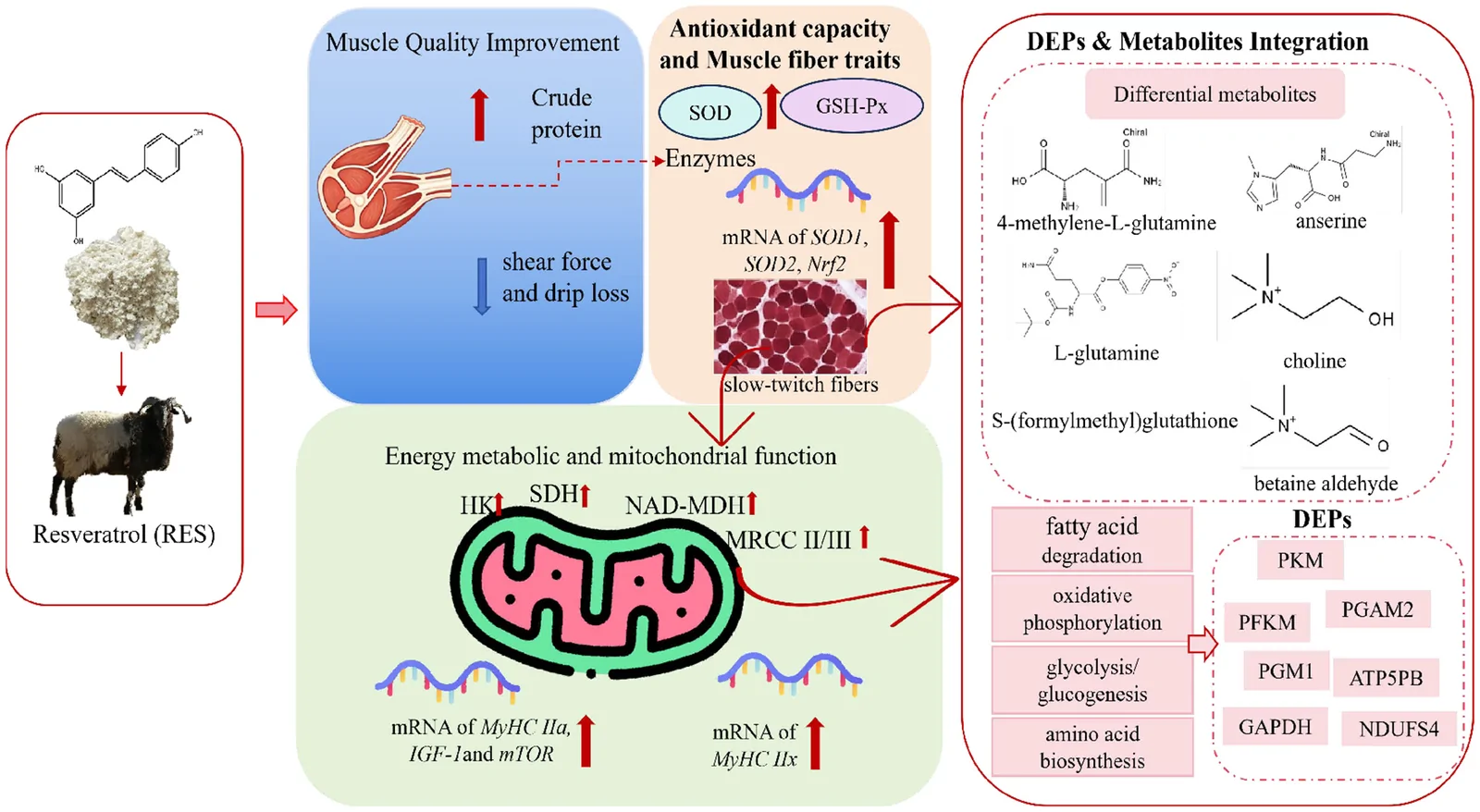
Schematic diagram of the regulatory mechanisms through which dietary RES improves meat quality in Tibetan sheep.

In this study, dietary RES supplementation enhanced multiple muscle phenotypes in Tibetan sheep, including improved meat quality, antioxidant capacity, muscle fiber composition, and metabolic enzyme activities. Mechanistically, elevated glucose content supplied sufficient substrates for glycolysis. Coupled with higher MRCC II/III activity, upregulated expression of *NRF1* and *TFAM*, and increased SDH and MDH activity, activated glycolysis synergistically strengthened mitochondrial oxidative metabolism. This coordinated energy supply meets the high energy demands of oxidative muscle fibers and facilitates fiber transformation toward the oxidative phenotype. Collectively, RES remodels glycolysis, fatty acid oxidation and mitochondrial respiratory pathways to optimize energy substrate distribution, coordinately modulating mitochondrial function, myofiber remodeling and antioxidant capacity, and ultimately achieving these phenotypic improvements. The complete regulatory cascade is illustrated in our mechanistic schematic diagram (Figure 7).

Although our findings provide a theoretical basis for the application of RES as a feed additive in plateau sheep production, certain limitations remain. This experiment was conducted under stall-feeding conditions, whereas Tibetan sheep are primarily grazed on natural pastures; therefore, the applicability of RES in grazing systems remains to be verified. Furthermore, the molecular mechanisms by which RES improves meat quality in Tibetan sheep have not been fully elucidated. Accordingly, in future studies, we will conduct an in-depth analysis of the molecular mechanisms by which RES improves Tibetan sheep meat quality at the cellular and genetic levels, and evaluate the practical efficacy of RES under natural grazing conditions.

## 6 Conclusions

Dietary RES supplementation improved meat quality (reduced shear force and drip loss, increased crude protein content), muscle antioxidant capacity (elevated SOD and GSH-Px activities, upregulated *Nrf2, SOD1*, and *SOD2*, and downregulated *Keap1* expression), muscle fiber characteristics (upregulated *MyHC IIa* and downregulated *MyHC IIx* expression, accompanied by an increased proportion of slow-twitch fibers), and metabolic enzyme activities (enhanced HK, SDH, and NAD-MDH activities) in Tibetan sheep. Metabolomic and proteomic analyses revealed that key proteins (PKM, PFKM, PGM1, PGAM2, HADHB, NDUFA8, NDUFS4, and GOT2) and differential metabolites (4-methylene-L-glutamine, N1-Methyl-2-pyridone-5-carboxamide, anserine, betaine aldehyde) were closely correlated with muscle antioxidant capacity, muscle fiber types, and mitochondrial function, jointly mediating the regulation of muscle oxidative homeostasis and meat quality. Furthermore, the higher RES dose (360 mg/kg) exerted more pronounced effects on muscle antioxidant capacity, meat color stability, and metabolic improvement in Tibetan sheep.

In conclusion, RES optimizes the allocation of energy substrates by upregulating key glycolytic proteins and integrating the three metabolic pathways of glycolysis, fatty acid oxidation, and mitochondrial respiration. This synergistically regulates muscle fiber type transformation and antioxidant capacity, ultimately improving meat quality. These findings provide a theoretical basis for the application of RES in the production of plateau Tibetan sheep.

## Data Availability

The datasets presented in this study can be found in online repositories. The names of the repository/repositories and accession number(s) can be found in the article/ Supplementary material.
