# Supplementary material for: Dietary resveratrol improves meat quality and antioxidant capacity of Tibetan sheep by regulating muscle fiber characteristics and mitochondrial function
Source: Front Nutr. 2026 Aug 14;13:1905065. doi: 10.3389/fnut.2026.1905065 (PMC13521774; doi:10.3389/fnut.2026.1905065)
Supplement: Supplementary file 1 [file Image_1.pdf]

Supplementary materials

Dietary Resveratrol improves meat quality and antioxidant capacity of Tibetan sheep by regulating muscle fiber characteristics and mitochondrial function

Xiongxiong Li[a,b], Zengkui Lu[a,b], Chao Yuan[a,b], Chen Zheng[c], Ting Liu[c], Bowen Chen[a,b], Jing Wang[a,b] and Jianbin Liu[a,b*]

[a] Key Laboratory of Animal Genetics and Breeding on Tibetan Plateau, Ministry of Agriculture and Rural Affairs, Lanzhou Institute of Husbandry and Pharmaceutical Sciences of Chinese Academy of Agricultural Sciences, Lanzhou, 730050 China
[b] Sheep Breeding Engineering Technology Research Center of Chinese Academy of Agricultural Sciences, Lanzhou, 730050 China
[c] College of Animal Science and Technology, Gansu Agricultural University, Lanzhou 730070, China.

*Corresponding Author: Jianbin Liu
Jianbin Liu Tel: 0931-2115273, E-mail: liujianbin@caas.cn

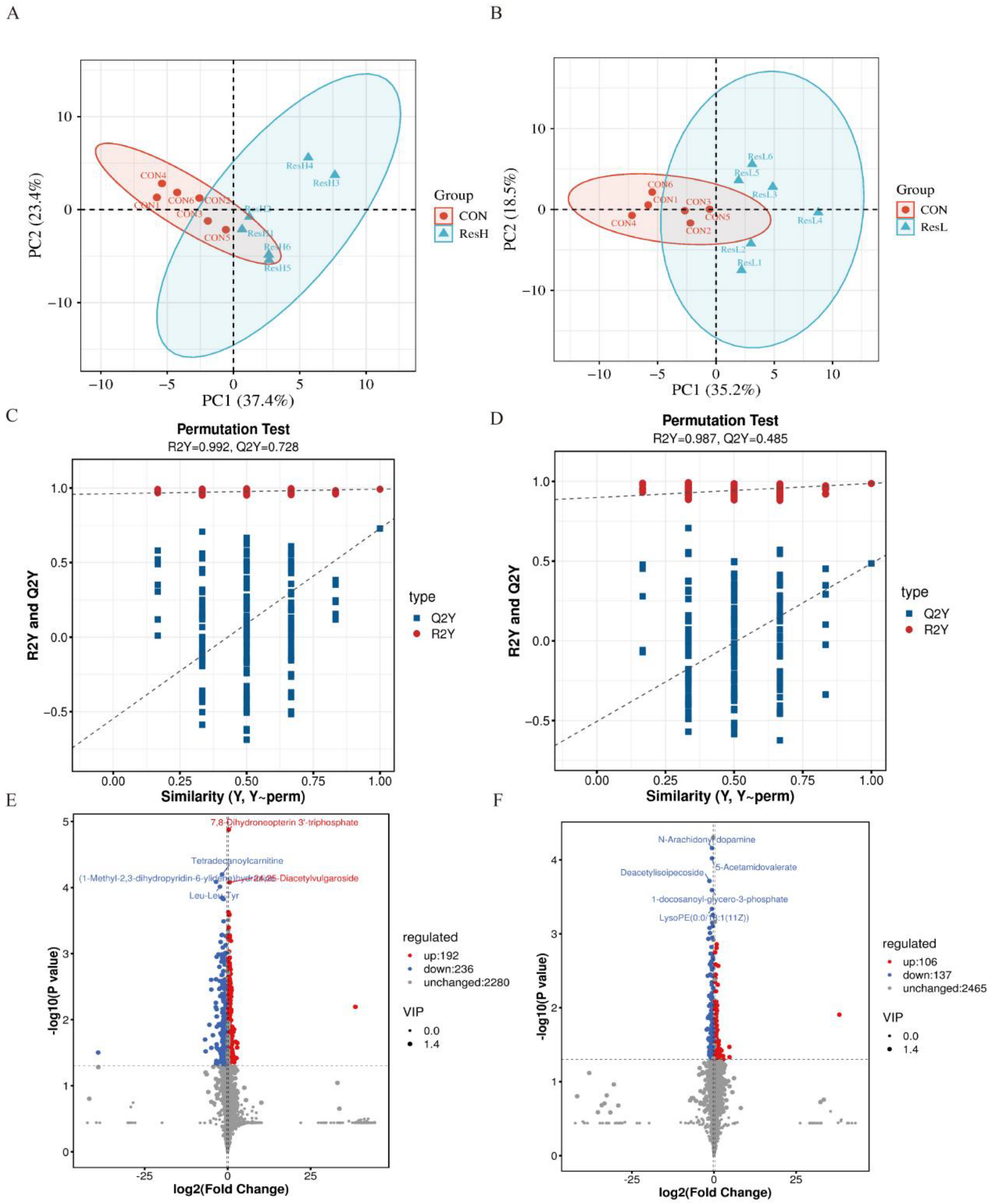

Fig. S1 Metabolic spectrum analysis. (A) PCA analysis between CON and ResH; (B) PCA analysis between CON and ResH; (C) Permutation testing model between CON and ResH; (D) Permutation testing model between CON and ResL; (E) Volcanic maps between CON and ResH; (F) Volcanic maps between CON and ResL.

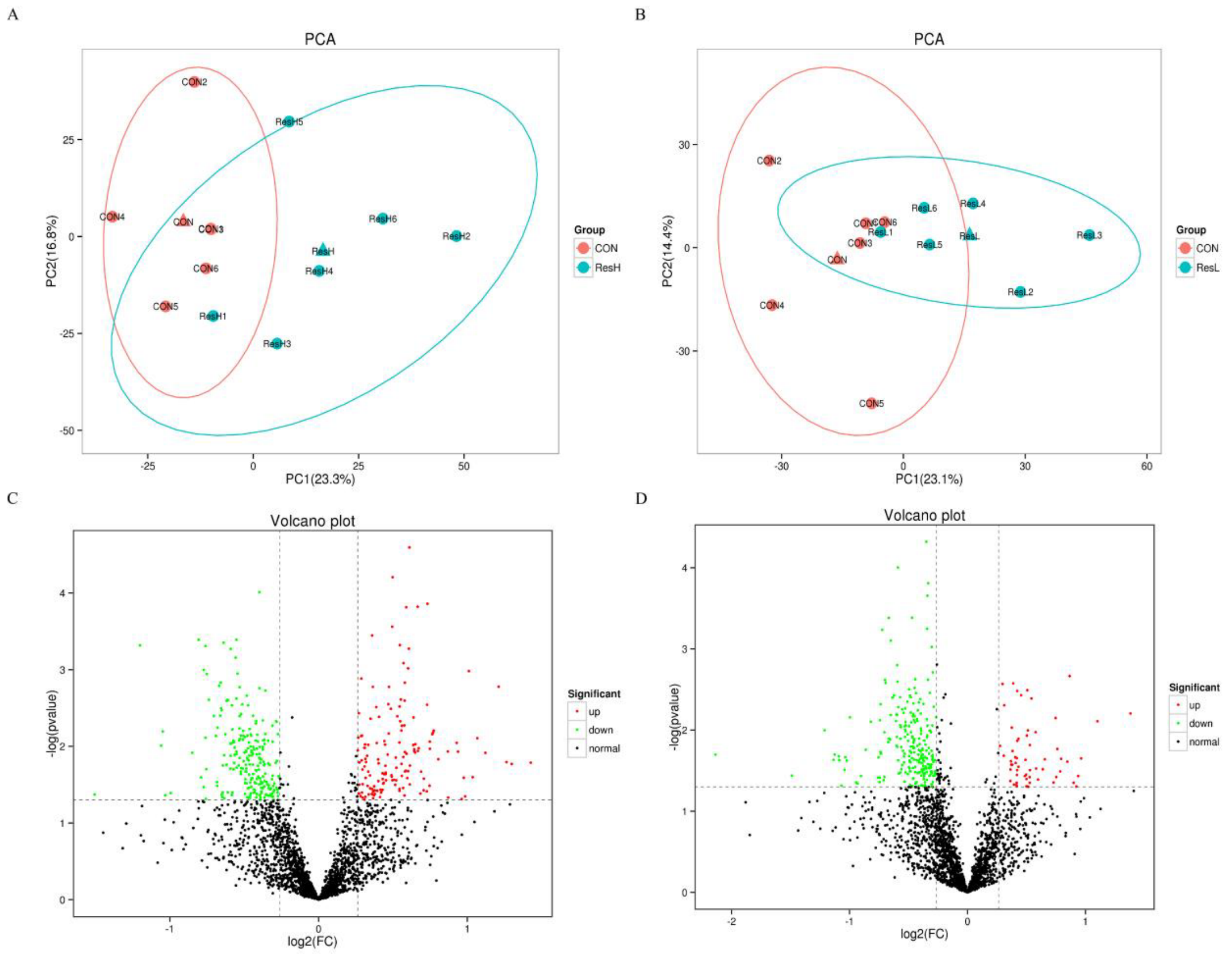

Fig. S2 DIA quantitative proteomics PCA and volcano plot. (A) PCA analysis between CON and ResH; (B) PCA analysis between CON and ResH; (C) Volcanic maps between CON and ResH; (D) Volcanic maps between CON and ResL.
